# Exploring Synergistic Interactions between Natural Compounds and Conventional Chemotherapeutic Drugs in Preclinical Models of Lung Cancer

**DOI:** 10.3390/ph17050598

**Published:** 2024-05-08

**Authors:** Mihaela Boța, Lavinia Vlaia, Alex-Robert Jîjie, Iasmina Marcovici, Flavia Crişan, Cristian Oancea, Cristina Adriana Dehelean, Tudor Mateescu, Elena-Alina Moacă

**Affiliations:** 1 Department II—Pharmaceutical Technology, Faculty of Pharmacy, “Victor Babeş” University of Medicine and Pharmacy, 2nd Eftimie Murgu Square, RO-300041 Timisoara, Romania; mihaela.rif18@gmail.com (M.B.); vlaia.lavinia@umft.ro (L.V.); 2 Formulation and Technology of Drugs Research Center, “Victor Babeş” University of Medicine and Pharmacy, 2nd Eftimie Murgu Square, RO-300041 Timisoara, Romania; 3 Department of Toxicology, Drug Industry, Management and Legislation, Faculty of Pharmacy, “Victor Babeş” University of Medicine and Pharmacy Timisoara, 2nd Eftimie Murgu Square, RO-300041 Timisoara, Romania; iasmina.marcovici@umft.ro (I.M.); flavia.crisan@umft.ro (F.C.); cadehelean@umft.ro (C.A.D.); alina.moaca@umft.ro (E.-A.M.); 4 Research Centre for Pharmaco-Toxicological Evaluation, “Victor Babeş” University of Medicine and Pharmacy, 2nd Eftimie Murgu Square, RO-300041 Timisoara, Romania; 5 Discipline of Pneumology, Department of Infectious Diseases, “Victor Babeș” University of Medicine and Pharmacy Timisoara, 2nd Eftimie Murgu Square, RO-300041 Timisoara, Romania; oancea@umft.ro; 6 Department of Thoracic Surgery, Clinical Hospital for Infectious Diseases and Pneumophthiology Dr. Victor Babes, 13 Gheorghe Adam Street, RO-300310 Timisoara, Romania; tudor.mateescu@umft.ro

**Keywords:** lung cancer, natural compounds, phytochemicals, medicinal plants, secondary metabolites, chemotherapy, synergistic interactions, combination therapy, preclinical models, therapeutic strategies

## Abstract

In the current work, the synergy between natural compounds and conventional chemotherapeutic drugs is comprehensively reviewed in light of current preclinical research findings. The prognosis for lung cancer patients is poor, with a 5-year survival rate of 18.1%. The use of natural compounds in combination with conventional chemotherapeutic drugs has gained significant attention as a potential novel approach in the treatment of lung cancer. The present work highlights the importance of finding more effective therapies to increase survival rates. Chemotherapy is a primary treatment option for lung cancer but it has limitations such as reduced effectiveness because cancer cells become resistant. Natural compounds isolated from medicinal plants have shown promising anticancer or chemopreventive properties and their synergistic effect has been observed when combined with conventional therapies. The combined use of an anti-cancer drug and a natural compound exhibits synergistic effects, enhancing overall therapeutic actions against cancer cells. In conclusion, this work provides an overview of the latest preclinical research on medicinal plants and plant-derived compounds as alternative or complementary treatment options for lung cancer chemotherapy and discusses the potential of natural compounds in treating lung cancer with minimal side effects.

## 1. Introduction

Lung cancer (LC) stands as the foremost cause of cancer-related deaths globally, primarily due to its often asymptomatic nature and detection in advanced stages when metastases to other vital organs are already present [1]. The 5-year survival rate for patients with LC is 18.1%, which is significantly lower than the average rate for patients with other types of cancer. This prognosis is discouraging for those diagnosed with this condition and more effective optimized therapies need to be found to increase survival rates [2]. Changes in smoking habits, advancements in the comprehension of lung cancer genetics, the role of the immune system in controlling lung cancer, and the array of treatment options have brought about notable shifts in the epidemiology and prevention of lung cancer in the past decade [3].

Depending on the stage of the disease, primary cancer treatment may include surgery, chemotherapy, radiotherapy, or targeted therapy [4]. Chemotherapy is a form of cytotoxic therapy that interferes with fundamental cellular functions, including cell proliferation, maintenance, metastasis, angiogenesis, and apoptosis, affecting not only cancerous cells but also normal ones. The effectiveness of chemotherapy is attributed to the fact that cancer cells tend to rely more heavily on these processes compared to healthy cells. Chemotherapy reduces the size of lung tumors, ameliorates cancer-related symptoms, extends patients’ longevity, and improves the chances of curing the disease when it is used alongside local treatments, like surgery or radiation. Additionally, it has the potential to boost the effectiveness of immune checkpoint inhibitors by modulating the immune system [5].

Phytomedicines are currently used in about 60% of cancer treatment strategies [6] and reports from the National Cancer Institute, which has evaluated over 35,000 plant species for potential anticancer properties, indicate that over 3000 plant species have promising anticancer or chemopreventive properties [4,7]. Bioactive compounds isolated from medicinal plants are key components in the therapeutic approach of cancer because their synergistic effect has been observed both in classical and conventional therapies [8]. Several phytomedicines have displayed anti-tumorigenic properties across various cancer types, including lung cancer [9]. For instance, *Ephedra alata* has been frequently used for the management of lung cancer in Palestine [10]. Additionally, *Rhus verniciflua* has shown therapeutic potential against lung cancer [11]. *Rheum officinale* has also been identified as a potential phytomedicine for inhibiting the progression of lung cancer [12]. Furthermore, combinations of natural products with chemotherapy drugs have been utilized in clinical trials against lung cancer, particularly non-small-cell lung cancer [13,14]. Due to its synergistic anticancer effects, lower toxicity from multiple mechanisms of action, and greater clinical application and perspective than monotherapy, drug–herbal combination therapy presents a viable prospect for cancer treatment. Additionally, combination therapy increases survival rates, significantly reduces the likelihood of cancer cells becoming resistant by increasing their sensitivity, and improves patients’ quality of life [14,15,16,17,18]. Phytocompounds are involved in the main pathways required for cancer initiation, progression, and metastasis, such as the cell cycle, DNA repair, apoptosis, or cell signaling [19,20]. Moreover, they also target pro-inflammatory proteins and reactive oxygen species, inhibit pro-angiogenic factors, regulate enzyme biotransformation, and modulate antimetastatic proteins [21]. It is estimated that a total of USD 5 billion is spent annually on the production of herbal anti-cancer drugs in Europe alone [4]. Natural compounds demonstrate potential efficacy in LC treatment, with minimal side effects [22]. The combined use of an anti-cancer drug and a natural compound exhibits synergistic effects, enhancing overall therapeutic actions against cancer cells. Various natural compounds can specifically target different cell signaling pathways linked to cancer progression, exerting a cytotoxic effect on the target cells [22,23,24,25]. For instance, studies have demonstrated the potential efficacy of compounds such as sulforaphane, apigenin, quercetin, and gallic acid in reducing cell viability and glycolysis of lung cancer cells [26]. Additionally, natural compounds like ellagic acid, myricetin, and polydatin have exhibited anti-lung cancer activity by inhibiting cell proliferation and inducing apoptosis [27,28,29]. Furthermore, oleuropein has been found to induce mitochondria-mediated apoptosis in lung cancer cells, suggesting its potential application in lung cancer treatment [30]. Moreover, compounds such as rabdocoetsin B, icariin, and ethoxysanguinarine have shown the ability to inhibit proliferation, induce apoptosis, and downregulate key proteins associated with lung cancer progression [31,32,33]. These findings highlight the potential of natural compounds in targeting various pathways involved in lung cancer development and progression. Furthermore, the use of natural compounds as chemosensitizing agents has been recognized as an important strategy for enhancing the efficacy of conventional chemotherapeutic drugs in lung cancer treatment [24]. Overall, natural compounds offer a promising avenue for the development of effective and minimally toxic treatments for lung cancer.

Based on this scientific background, the present work provides a comprehensive overview of the latest research status of medicinal plants and plant-derived compounds as alternative or complementary treatment options to LC chemotherapy. The paper aims to provide a new approach to the treatment of lung cancer, starting with a brief description of the pathological features of lung malignant neoplasms and the available chemotherapeutics for their treatment. The paper outlines the most exploited plant species and phytocompounds for LC management and provides a glance at their potential synergistic interaction with the marketed anti-tumor agents used in LC therapy.

## 2. Pathological Characteristics of Lung Cancer

In recent years, progress in LC has been significantly propelled by a deeper understanding of the utilization of predictive biomarkers, refinements in treatment modalities, and the disease’s biology. These advancements have resulted in substantial improvements and good outcomes for many patients but, with all this, the number of patients diagnosed with lung cancer (particularly in low-income countries with limited access to healthcare) continues to rise [34].

The causation of lung cancer is a multifactorial process influenced by genetic, environmental, lifestyle, and molecular factors. Tobacco smoking, exposure to asbestos, air pollution, and genetic predisposition are among the prominent risk factors for lung cancer. Tobacco smoking is a major cause of lung cancer and the combined effect of smoking and asbestos exposure is greater than the sum of the individual effects [35,36]. Alterations in the expression of microRNA genes have been found to contribute to the pathogenesis of lung cancer, indicating the involvement of genetic factors in the development of the disease [37]. Studies have also shown that lung cancer with TP53 alterations carries a worse prognosis and may be relatively more resistant to chemotherapy and radiation, highlighting the role of genetic mutations in the progression and treatment response of lung cancer [38]. Environmental factors such as exposure to polycyclic aromatic hydrocarbons (PAHs) from tobacco smoke and air pollution have been implicated in the causation of lung cancer. Evidence exists to support the metabolic activation of PAHs in humans, indicating a direct link between environmental pollutants and the development of lung neoplasms [39]. The role of the microbiome in the pathogenesis of lung cancer has also been suggested, although the underlying mechanisms are still poorly understood [40]. Lifestyle choices and molecular mechanisms also contribute to the causation of lung cancer. Carcinogens present in tobacco products and their intermediate metabolites can activate multiple signaling pathways that contribute to lung cancer carcinogenesis [41]. Additionally, specific microRNAs, such as miR-150, have been implicated in promoting the proliferation of lung cancer cells by targeting P53, indicating the involvement of molecular regulatory mechanisms in the development of the disease [42]. Understanding these diverse influences is crucial for developing effective strategies for the prevention, diagnosis, and treatment of lung cancer.

LC represents the leading cause of cancer-related deaths worldwide, with a mortality rate of 1.80 million and an incidence of 2.24 million cases per year. This prognosis for individuals with LC is intricately tied to the stage at which the cancer is diagnosed: stage I (tumors ≤ 3 cm) exhibit a five-year survival rate of 68.4%, in contrast to those diagnosed with stage IV, who face a considerably lower five-year survival rate of 5.8% [43]. Unfortunately, a significant portion of LC cases are detected at stage IV, contributing to a diminished survival outlook and heightened symptom severity. In addition to the cancer stage, the histological classification is also important in establishing the mortality rate. Lung cancer can be divided into two primary histological groups: small cell lung carcinoma (SCLC), which accounts for 15% of all lung cancers, and non-small cell lung cancer (NSCLC), which represents 85% of all lung cancers. NSCLCs are further classified into subtypes such as squamous cell carcinoma (SqCC), large cell carcinoma (LCC), and adenocarcinoma [44]. The risk of LC is reportedly higher in men than in women [45].

The incidence of lung adenocarcinoma has been steadily rising in recent decades, likely attributed to the widespread use of low-nicotine-tar cigarettes. This trend is more noticeable in male patients but can also be found in women, the young, and those who have never smoked. Invasive adenocarcinoma is categorized based on the predominant pattern of growth exhibited by the neoplastic cells, which include lepidic, acinar, papillary, micropapillary, and solid patterns. Additionally, there are four recognized variants of invasive adenocarcinoma, invasive mucinous, colloid, fetal, and enteric [46], and the preliminary stages encompass two entities: adenocarcinoma in situ (AIS) and atypical adenomatous hyperplasia (AAH) [47]. The classification of lung squamous cell carcinoma (SqCC) involves categorizing it into keratinizing, non-keratinizing, and basaloid subtypes [48]. In large cell carcinoma (LCC), the diagnosis of one of the three major histologic subtypes of non-small cell lung cancer (NSCLC) relies significantly on immunophenotypic analysis. Following immuno-histochemical (IHC) examination, undifferentiated NSCLC cases that present as solid adenocarcinoma or non-keratinizing squamous cell carcinoma exhibiting null or unclear immunophenotypes are presently categorized as LCC [49].

Small-cell lung cancer (SCLC) tends to disseminate early, leading to the diagnosis of extensive disease in many patients. Despite chemotherapy, the majority of patients experience relapse, with rare survival beyond 2 years [50]. Genomic profiling of SCLC exposes widespread chromosomal rearrangements and a high mutation burden, often involving the functional inactivation of the tumor suppressor genes TP53 and RB1 [51]. The approach to treating lung cancer depends on factors such as the cancer type, its extent of spread, and the individual’s medical history. Treatment modalities encompass surgical procedures, radiotherapy, targeted therapy, immunotherapy, and chemotherapy [52].

The pathogenic mechanisms of lung cancer can vary between different age groups, ethnicities, and disease states. Studies have shown that there are racial and ethnic differences in the epidemiology and genomics of lung cancer, with higher incidence rates and lower survival rates observed in certain ethnic groups, such as in black people compared to white people [53,54]. Furthermore, research has demonstrated that there are differences in genetic risk and smoking as well as their interactions with lung cancer between different cohorts, including the China Kadoorie Biobank and the UK Biobank [55].

Age also plays a crucial role in the pathogenesis of lung cancer. Comprehensive comparative molecular characterization of young and old lung cancer patients has revealed differences in the molecular profiles of tumors between these age groups, indicating potential age-related variations in the pathogenic mechanisms of lung cancer [56]. Additionally, COPD has been identified as a disease of immunosenescence, with aging contributing to the development of inflammation in the lungs, which may lead to COPD and potentially increase the risk of lung cancer [57].

Moreover, the role of the lung microbiome in the innate immune response associated with chronic lung diseases, including lung cancer, has been investigated. This highlights the potential impact of disease states, such as chronic lung diseases, on the pathogenic mechanisms of lung cancer [58]. Furthermore, the contribution of hereditary cancer-related germline mutations to lung cancer susceptibility has been studied, revealing the significance of genetic factors in the pathogenesis of lung cancer [59].

In addition to age, ethnicity, and disease states, genetic predisposition associated with inflammatory response has been implicated in the development of COPD, which is a risk factor for lung cancer [60]. Furthermore, the co-occurrence of pulmonary fibrosis and lung cancer, associated with germline mutations, is linked to worse survival outcomes, emphasizing the intricate interplay between different disease states in the pathogenesis of lung cancer [61].

These variations encompass a wide range of factors, including genetic predisposition, inflammatory response, and the interplay between different disease states, highlighting the complexity of lung cancer pathogenesis.

## 3. Chemotherapy for Lung Cancer Treatment

### 3.1. Current Chemotherapeutic Drugs Used in Lung Cancer Treatment

In the 1970s, early-generation chemotherapeutic drugs like methotrexate and doxorubicin had limited clinical impact on lung cancer treatment. Notable advancements in patient survival occurred in the 1980s and 1990s with the introduction of platinum-based and more advanced medications such as taxanes, vinorelbine, and gemcitabine. In the early 2000s, pemetrexed emerged as an effective new-generation chemotherapy for lung cancer. Additionally, the mid-2000s to early 2010s saw the success of anti-angiogenesis drugs like bevacizumab and innovative drug delivery systems like nanoparticle albumin-bound paclitaxel (nab-paclitaxel), which were both approved as valuable agents for lung cancer treatment due to their clinical benefits in terms of efficacy and patient tolerance [62].

Over the past 30 years, significant progress in treating NSCLC has included the establishment of palliative chemotherapy for advanced cases, the adoption of adjuvant chemotherapy for individuals with fully resected NSCLC, and the recognition of chemotherapy as a primary treatment approach for patients with locally advanced NSCLC [63].

The primary treatment for unresectable advanced NSCLC involves cytotoxic anti-cancer agents, predominantly platinum-based drugs. Standard therapy consists of combination chemotherapy using two drugs, typically one platinum-based and one unrelated cytotoxic drug, applied across various histological types [64]. Recent years have seen the identification of genetic abnormalities, termed driver mutations, in NSCLCs, such as epidermal growth factor receptor (EGFR) mutations and anaplastic lymphoma kinase translocations, driving carcinogenesis and cancer progression. Molecular targeted therapies addressing these mutations have improved the NSCLC prognosis [65]. Monoclonal antibodies have emerged as versatile platforms for cancer immunotherapy, targeting specific antigens and complement system proteins to enhance the immune response against cancer cells [66]. For instance, panitumumab, a fully humanized IgG2 isotype antibody specific for EGFR, has shown efficacy in inhibiting cancer progression through mechanisms such as antibody-dependent cellular cytotoxicity (ADCC) [67]. Additionally, the use of monoclonal antibodies targeting HER2 mutations has shown promise in non-small cell lung cancer (NSCLC) treatment, particularly in cases where HER2 mutation is mutually exclusive with other driver mutations such as EGFR, ALK, ROS1, and BRAF [68]. Furthermore, the combination of immunotherapy, particularly monoclonal antibodies, with conventional chemotherapy has been a focus of extensive research and clinical trials. Studies have evaluated the addition of immunotherapy to conventional chemotherapy in patients with extensive small-cell lung cancer (SCLC), demonstrating potential benefits in terms of treatment efficacy and patient outcomes [69]. Additionally, the use of pembrolizumab, a monoclonal antibody targeting programmed cell death-1 (PD-1), has been approved as a first-line treatment for advanced NSCLC with robust PD-L1 expression, further highlighting the significance of monoclonal antibody-based therapies in improving survival rates [70]. Yet, effective targeted drugs for squamous cell carcinoma and a significant portion of adenocarcinomas remain undeveloped. The appropriate use of existing cytotoxic agents is crucial for treating patients with these specific cancer types [65].

Both metronomic chemotherapy and lurbinectedin, an RNA polymerase II inhibitor, hold promise in the treatment of small cell lung cancer (SCLC), with metronomic chemotherapy indicating enhanced survival despite significant toxicity concerns, while lurbinectedin shows promise pending validation from ongoing trials [71]. In recent years, combinations of chemotherapy with tyrosine kinase inhibitors (TKIs) or immune checkpoint inhibitors (ICIs) have shown improved survival outcomes when compared to chemotherapy alone [72].

Antibody–drug conjugates (ADCs) represent a promising approach in cancer treatment, aiming to deliver toxic drugs specifically to tumor cells that display particular antigens associated with malignancy. The fundamental components of an ADC consist of the antibody, cytotoxic agent, and linker. This innovative therapeutic strategy is anticipated to offer potent treatment options for diverse cancers by leveraging the targeted precision of monoclonal antibodies (mAbs) and the effectiveness of various chemotherapeutic agents. The combined action of these three elements forms a highly efficient anti-tumor agent, delivering chemotherapy drugs directly and selectively to cancer cells, guided by antibodies with remarkable specificity and affinity [73].

### 3.2. Limitations of Chemotherapy in Lung Cancer Treatment

Resistance to chemotherapy poses a significant challenge to effectively treating advanced lung cancer. Certain lung tumors inherently resist chemotherapy and, in almost all instances, individuals who initially respond to treatment quickly develop acquired resistance [74]. Mechanisms of chemotherapy resistance can be roughly classified into three types: changes to the targeted oncogenes through secondary mutations or amplification, activation of alternative oncogenic signaling pathways, and alteration of cellular traits. Overcoming resistance is a current and critical area of focus in clinical research [75].

Continued exploration into the molecular mechanisms of chemotherapy resistance and the identification of predictive biomarkers for chemotherapy sensitivity are crucial. A more enhanced comprehension of resistance mechanisms at the molecular level could provide avenues for combining traditional chemotherapeutic agents with molecularly targeted ones. This approach holds promise as a strategy to overcome chemotherapy resistance and enhance therapy optimization for individuals with lung cancer [74].

Another limitation of chemotherapy in lung cancer treatment is the KRAS mutation in lung cancer, which was identified in 1982 and is found in about 30% of lung adenocarcinomas in Caucasians and around 15% in east Asians. Despite its high occurrence and long history, there has not been a successful treatment specifically for cancers with KRAS mutations. This is likely due to two main reasons: first, it is challenging to target the KRAS mutation itself and second, some KRAS-mutated lung cancers can survive and grow without relying on the mutant KRAS gene. There is evidence suggesting that initially, KRAS mutations may be crucial for the survival of tumors but over time, they can become independent of KRAS function as the tumor develops [75]. In the treatment of lung cancer brain metastases in individuals who have already undergone chemotherapy or radiotherapy, the role of chemotherapy seems to be more limited [76].

## 4. Medicinal Plants and Phytocompounds with Anticancer or Chemopreventive Activity in Lung Cancer

Medicinal plants and phytocompounds isolated from these species have been used since ancient times in the treatment of various diseases, such as diabetes, cardiovascular diseases, or cancer, due to their multiple beneficial and curative effects [77]. Medicinal plants are a rich source of biologically active compounds, which are divided into primary and secondary metabolites [4].

Medicinal plants and phytocompounds have been extensively studied for their potential as antineoplastic agents in lung cancer. For lung cancer treatment, the antitumor effects of plants and phytocompounds involve various target mechanisms of action on tumor growth such as apoptosis, reduction in metastasis, and specific pathway modulation [19,20]. One such mechanism is the inhibition of cell proliferation and viability through the modulation of cell cycle regulators (for example, by inducing cell cycle arrest at the G1 phase), while another mechanism involves the induction of apoptosis by the modulation of apoptotic proteins (Bax and Bcl2) or activation of apoptotic signaling pathways (AXL phosphorylation, Akt/mTOR, PI3/AKT, and p-JNK) in lung cancer cells, which are crucial for cancer cell survival and proliferation [78,79,80]. Additionally, some medicinal plants and phytocompounds have been found to possess anti-inflammatory properties and can inhibit the development and progression of lung cancer [81]. Furthermore, some of the natural compounds have been shown to inhibit angiogenesis, which is crucial for tumor growth and metastasis [82]. These mechanisms collectively suggest that medicinal plants and phytocompounds have the potential to be effective in the treatment and prevention of lung cancer as alternative or complementary therapy strategies [83,84].

Numerous studies demonstrate the chemopreventive effect of phenolic compounds and following a diet rich in them can prevent cancer recurrence. They modulate oxidative stress and can initiate apoptosis in cancer cells by regulating the mobilization of copper ions that are bound to chromatin, leading to DNA fragmentation. Some compounds, such as flavonoids, tannins, curcumin, resveratrol, or galactacin, have demonstrated potent anti-cancer properties. From the class of phenolic compounds, flavonoids play a special role in cancer therapy [4]. The choice to investigate flavonoids stems from their established role in the complex mechanisms of histone deacetylase-mediated chromatin remodeling and the epigenetic modulation of gene expression. The inhibition of histone deacetylases has emerged as a strategic approach to induce specific epigenetic changes linked to cancer pathogenesis [85]. Flavonoids exhibit antioxidant, anti-inflammatory, and anti-cancer activities by targeting various cellular pathways involved in cancer development and progression. Notably, apigenin, a member of the flavonoid class, has demonstrated these effects, underscoring the heightened importance of this compound group in the field of epigenetic research [86,87]. The pivotal role of diet in various pathologies, including lung cancer, is widely acknowledged. Natural compounds, particularly flavonoids, exert a notable influence on chemotherapy interventions by mitigating adverse effects, counteracting acquired resistance, and enhancing the bioavailability of pharmaceutical agents [88].

Triterpenes manifest diverse therapeutic actions, including anti-inflammatory, antitumoral, and antiviral effects [89]. Their significance in the medical domain is particularly noteworthy, given that these properties have been scrutinized in conjunction with chemotherapy, resulting in a synergistic interplay. Amid the extensive exploration into the effects of triterpenes, considerable attention has been dedicated to the triterpenoid constituents of *Boswellia serrata*, *Lantana camara* [90], and *Hedera helix* [91].

Some phytocompounds, such as 6-gingerol, vitexin, nimbolide, ursolic acid, withaferin A, sulforaphane, and emodin, isolated from various plant species, have been tested in preclinical studies against lung cancer. Due to their promising results, they have been approved for the preliminary phase of clinical trials, along with other phytocompounds such as berberine, curcumin, epigallocatechin gallate, quercetin, and resveratrol [4].

The best-known plant species as generators of natural compounds with anti-cancer properties against lung cancer are *Taxus* sp. (for the extraction of diterpene alkaloids, collectively called taxanes, namely paclitaxel, docetaxel, and cabazitaxel), *Catharanthus roseus* (for the extraction of vincristine and vinblastine alkaloids), *Podophyllum peltatum* (for the extraction of the lignan podophyllin), and *Camptotheca acuminata* (for the extraction of camptothecin derivatives: topotecan) [4].

Table 1 provides a comprehensive summary of pertinent preclinical investigations demonstrating the advantageous impacts of medicinal plants and phytocompounds in the management of lung cancer. The table offers a detailed compilation of the various studies that have examined the therapeutic potential of these natural substances, shedding light on their promising effects in preclinical models of lung cancer.

### 4.1. Dryopteris erythrosora

A study that used an ethanolic extract from the leaves and rhizomes of the fern *Dryopteris erythrosora* on the A549 lung cancer cell line revealed potent free radical scavenging activity of the flavonoids in the extract and significant cytotoxicity against cancer cells [112].

### 4.2. Brassinosteroids

Brassinosteroids isolated from the pollen of *Brassica napus* (Brassicaceae) and other *Brassica* species demonstrated cytotoxic effects on cancer cells using the A549 lung cancer cell line [4,92].

### 4.3. Haemanthus humilis

The alkaloid compounds coccinine and montanine, isolated from the plant *Haemanthus humilis*, showed potent tumor cell growth inhibitory properties in the A549 lung cancer cell line [93].

### 4.4. Taxus *sp.*

Taxanes isolated from the bark, leaves, seeds, and roots of *Taxus* species (family Taxaceae) are used in the treatment of lung cancer due to their ability to bind to the beta-tubulin subunit of microtubules, stopping the polymerization-depolymerization processes of microtubules, which stabilize cancer cells, which enter apoptosis due to the impossibility of cell division [94].

### 4.5. Catharanthus roseus

Alkaloids from the aerial parts and roots of *Catharanthus roseus* (family Apocynaceae) are used in the treatment of LC because they block the cell cycle in metaphase, altering the functions of the microtubules necessary for the formation of the division spindle, with cancer cells entering apoptosis due to the stabilization of the microtubules [95].

### 4.6. Podophyllum peltatum

Podophyllotoxin and its semi-synthetic analogs (etoposides) extracted from the rhizomes of *Podophyllum peltatum* (family Berberidaceae) are used in the treatment of lung cancer due to the formation of complexes between the enzyme topoisomerase II and DNA, preventing the binding of DNA strands, which, through accumulation, lead to apoptosis of cancer cells [96].

### 4.7. Camptotheca acuminata

Camptothecin derivatives, such as topotecan, isolated from the bark and stem of *Camptotheca acuminata* (family Nyssaceae), can be used in the treatment of lung cancer due to the formation of ternary complexes by binding to the enzyme topoisomerase I and DNA, leading to double-stranded DNA damage and cancer cells entering apoptosis [97].

### 4.8. Panax ginseng

Ginsenoside Rg3, a steroidal saponin found in the roots of *Panax ginseng* (family Araliaceae), has demonstrated anticancer effects in lung cancer by inhibiting tumor migration and invasion through various mechanisms. Specifically, it has been found to suppress lung cancer migration and invasion by inhibiting TGF-β1-induced EMT, accompanied by the inactivation of MMP-2, p38 MAPK, and Smad2 [101]. Moreover, Ginsenoside Rg3 has been reported to attenuate the stemness of non-small cell lung cancer cells, reducing spheroid formation ability, ALDH1 activity, and stemness marker expression [100]. Additionally, it has been shown to enhance the antitumor activity of gefitinib in lung cancer cell lines by enhancing the expression levels of cell migration- and apoptosis-associated markers [99]. These findings collectively suggest that Ginsenoside Rg3 holds promise as a potential therapeutic agent in lung cancer treatment, with its ability to target multiple pathways involved in cancer progression and resistance [98].

### 4.9. Daphne genkwa

The phytocompound yuanhuacine (a daphnan-type diterpenoid), isolated from the flower buds of the plant *Daphne genkwa* (family Thymelaeaceae), showed potent growth inhibitory activity against non-small cell lung cancer (NSCLC) tumor cells in the study by Kang et al. The NSCLC cell line H1993 and an in vivo nude mouse xenograft model with implanted H1993 cells were used. Yuanhuacine demonstrated selective growth inhibitory activity on human lung cancer cells compared to normal lung epithelial cells, with its antiproliferative activity being concentration and time-dependent. Mechanistic studies revealed that yuanhuacine treatment led to increased AMP-activated protein kinase (p-AMPK) levels and decreased p-acetyl-CoA carboxylase (ACC) levels after 24 h, implicating AMPK as a potential target in yuanhuacine’s antiproliferative activity. Furthermore, yuanhuacine treatment suppressed F-actin expression, inhibited cancer cell migration and invasion, and exhibited a synergistic inhibitory effect when combined with the chemotherapeutic drugs gefitinib and rapamycin. Additionally, in a nude mouse xenograft model (0.5–1 mg/kg, once daily), yuanhuacine significantly reduced tumor growth and weight, possibly through the modulation of AMPK and mTOR signaling pathways [102].

### 4.10. Aromatic Plants

The phytocompound carvacrol (a monoterpenic compound), mainly isolated from the volatile oils of aromatic plants (such as *Origanum vulgare* and *Thymus vulgaris*, family Lamiaceae), showed favorable effects in the treatment of non-small cell lung cancer (NSCLC) in the study by Jung et al. The treatment of non-small cell lung cancer (NSCLC) with concentrations between 100 and 300 μM carvacrol resulted in reduced AXL expression and phosphorylation in A549 and H460 cell lines. This reduction occurred at both the transcriptional and protein levels in a dose-dependent and time-dependent manner. Carvacrol treatment also led to decreased cell viability, inhibition of AXL phosphorylation induced by GAS6, and reduced cell colony formation and migration in both cell lines. These findings suggest that carvacrol has promising effects on AXL expression and activation in NSCLC, indicating its potential for the treatment of this type of lung cancer [103].

### 4.11. Tripterygium wilfordii

The phytocompound celastrol (a pentacyclic nortriterpenquinone), isolated from the roots of *Tripterygium wilfordii* (family Celastraceae), demonstrated pro-apoptotic properties in the A549 human lung adenocarcinoma cell line in the study by Yan et al. Celastrol inhibited lung cancer cell proliferation and promoted apoptosis in a dose-dependent manner by reducing the phosphorylation levels of STAT3 and the Bcl-2/Bax ratio. It also modulated the expression levels of miR-24 and miR-181b, leading to the suppression of cancer cell proliferation, inhibition of invasion, and prevention of angiogenesis. Treatment with varying concentrations of celastrol on cell lines resulted in dose-dependent inhibition of cell proliferation and induction of apoptosis, with more pronounced effects observed at higher concentrations (3 μM) [104].

### 4.12. Lycoris radiata

The phytocompound lycorine (an isoquinoline alkaloid), isolated from the bulbs of the plant *Lycoris radiata* (family Amaryllidaceae), showed favorable effects in the treatment of non-small cell lung cancer (NSCLC) using A549 and H460 cell lines (in vitro) and an A549/Luc tumor xenograft nude mouse model (in vivo) in the study by Sun et al. Lycorine was found to inhibit the growth of NSCLC cells, arrest the cell cycle in the G0/G1 phase, and trigger apoptosis through the caspase-mediated signaling pathway. Additionally, lycorine maintained cellular levels of β-catenin and GSK-3b, decreased the expression of mesenchymal marker N-cadherin, and reduced the expression of cytoskeletal protein F-actin. In a tumor xenograft nude mouse model, lycorine treatment (2.5 mg/kg body weight and 5.0 mg/kg body weight) inhibited tumor growth and metastasis, leading to prolonged survival compared to cisplatin-treated groups, without observable toxicity reactions [105].

### 4.13. Ginkgo biloba

The phytocompound ginkgolic acid C15:1 (phenolic acids class), isolated from the leaves and pseudo-drupes of *Ginkgo biloba* (family Ginkgoaceae), demonstrated favorable effects in controlling metastatic dissemination of lung cancer tumor cells by regulating the epithelial–mesenchymal transition (EMT) pathway in the study by Baek et al. The treatment with GA C15:1 at concentrations ranging from 30 μM to 150 μM resulted in dose-dependent cytotoxic effects, inhibition of cell proliferation, invasion, and migration. GA C15:1 also inhibited the genes and markers associated with the epithelial–mesenchymal transition (EMT) pathway in lung cancer cells. Furthermore, it reduced TGF-β-induced morphological changes and suppressed tumor cell invasion and migration by decreasing the phosphorylation of the PI3K/Akt/mTOR signaling pathways. Additionally, GA C15:1 treatment led to a significant reduction in Akt activation in a dose-dependent manner, thereby inhibiting lung cancer cell migration and invasion [106].

### 4.14. Rosmarinus officinalis

The phytocompounds carnosic acid (a phenolic diterpene) and rosmarinic acid (a phenolic compound), extracted from the leaves of *Rosmarinus officinalis* (family Lamiaceae), may offer a good strategy for the treatment of chemotherapy-resistant lung cancer, according to a study by Yesil-Celiktas et al. The extract was tested on the NCI-H82 human small-cell lung carcinoma cell line at concentrations ranging from 6.25 to 100 μg/mL. Carnosic acid demonstrated superior results, achieving the lowest cell viability at a concentration of 6.25 μg/mL, with values ranging from 13 to 30%. The mechanism of carnosic acid-induced tumor cell apoptosis involves cell cycle arrest in the G2/M phase at various stages. In contrast, rosmarinic acid exhibited more significant proliferative effects than cytotoxic activity, with 205% cell viability at 139 μM (50 mg/mL). The average mean inhibitory concentration (IC_50_) for the NCI-H82 cell line was 56.40 μg/mL [107].

### 4.15. Ophiopogon japonicus

The phytocompound ophiopogonin D (a steroid glycoside), isolated from the roots of the plant *Ophiopogon japonicus* (family Asparagaceae), can inhibit various signaling cascades related to tumorigenesis in lung cancer and can be used in combination therapy with chemotherapeutic agents, as indicated by the results of studies by Lee et al. [108]. Ophiopogonin D has been shown to inhibit proliferation, cell adhesion, and invasion in lung cancer cell lines H1299 and A549, inducing apoptosis. It also inhibits the activation of NF-κB, PI3K/AKT, and AP-1 signaling pathways, reducing the expression of oncogenic gene products. Ophiopogonin D demonstrated selectivity in producing cytotoxic effects only on tumor cells, as revealed by the MTT assay comparing its effects on lung cancer cell line A549 and human lung non-cancer cell line HEL 299. It was also found that ophiopogonin D and paclitaxel have potent synergistic effects in inducing cancer cell apoptosis. The combination of sub-optimal doses of both inhibited cell proliferation and induced apoptosis, potentiating each other’s effects [108].

### 4.16. Sphagneticola calendulacea

The major phytocompounds carvacrol and trans-caryophyllene, isolated from the volatile oil extracted from the leaves of the plant *Wedelia chinensis*, renamed *Sphagneticola calendulacea* (family Asteraceae), were evaluated in vivo in the study by Manjamalai et al. to determine their effects in the treatment of lung cancer. The volatile oil containing carvacrol and trans-caryophyllene exhibited dose-dependent free radical scavenging capacity, with maximum DPPH radical scavenging of 65.91% and hydroxyl radical scavenging of 68.04% at 100 μg/mL. The IC_50_ values for DPPH and hydroxyl radical scavenging were 48 μg/mL and 51 μg/mL, respectively, indicating potent antioxidant activity. In a C57BL/6 mouse model with B16F10 melanoma tumor xenografts, administration of volatile oil at doses ranging from 10 to 100 μg resulted in a significant decrease in tumor mass, regeneration of alveolar passage, and normalization of lung tissue. The treatment also led to reduced malondialdehyde levels, increased glutathione peroxidase activity, and elevated superoxide dismutase and catalase levels, demonstrating its potent antioxidant activity in vivo [109].

### 4.17. Dendrobium *sp.*

The phytocompound chrysotobibenzyl, isolated from the stems of the orchid species *Dendrobium pulchellum* (family Orchidaceae), shows an inhibitory effect on lung cancer cell migration by suppressing caveolin-1 (CAV-1) activity, integrins, and the epithelial-to-mesenchymal transition (EMT), according to the study by Petpiroon et al. Other compounds isolated from species of the genus *Dendrobium* also have anti-metastatic effects in the treatment of lung cancer, such as giganthol (*D. draconis*), chrysotoxins, crepidatin, and moscatilin (*D. pulchellum*). Treatment with concentrations ranging from 1 to 50 µM chrysotobibenzyl resulted in dose-dependent inhibition of cancer cell migration and invasion, filopodia formation, and decreased EMT of human NSCLC lines H460, H292, A549, and H23. Additionally, chrysotobibenzyl sensitized lung cancer cells to cisplatin-mediated apoptosis and exhibited anti-migratory effects stronger than cisplatin at a similar concentration. Furthermore, chrysotobibenzyl demonstrated mild cytotoxicity at 100 µM and significant cell disruption at 50 µM in certain cell lines [110].

### 4.18. Citrus aurantium and Matricaria recutita

The phytocompounds apigenin and naringenin are flavonoids isolated from various plants, such as *Citrus aurantium* (family Rutaceae) or *Matricaria recutita* (family Asteraceae) [113], whose individual and synergistic effects in cancer treatment have been studied and shown to have promising results. In the study by Liu et al., the effects of apigenin (Api) and naringenin (Nar) and the combination of the two compounds (Api-Nar) were studied on A549 and H1299 NSCLC cell lines. The combination of apigenin and naringenin has demonstrated synergistic antiproliferative effects in lung cancer cell lines. The optimal mixture ratio is 3:2. This combination is more potent in reducing cell numbers and inducing changes in cell morphology. It disrupts cell cycle progression and increases apoptotic rates, Caspase-3 activity, and levels of pro-apoptotic Bax proteins. It also elevates malonaldehyde (MDA) concentration and reactive oxygen species (ROS) levels and decreases mitochondrial membrane potential and ATP synthesis, ultimately promoting tumor cell apoptosis [111].

Table 2 presents the chemical structures of the primary phytocompounds derived from various medicinal plants that have exhibited significant therapeutic potential in the management of lung cancer based on preclinical investigations. These phytocompounds, sourced from diverse botanical origins, have demonstrated promising anti-cancer properties through their interactions with key molecular targets involved in tumorigenesis and cancer progression. By elucidating the chemical structures of these bioactive compounds, Table 2 offers a comprehensive insight into the molecular basis of their pharmacological activities, thereby laying a solid foundation for further research endeavors aimed at harnessing their therapeutic benefits for lung cancer patients.

**Figure 1:**
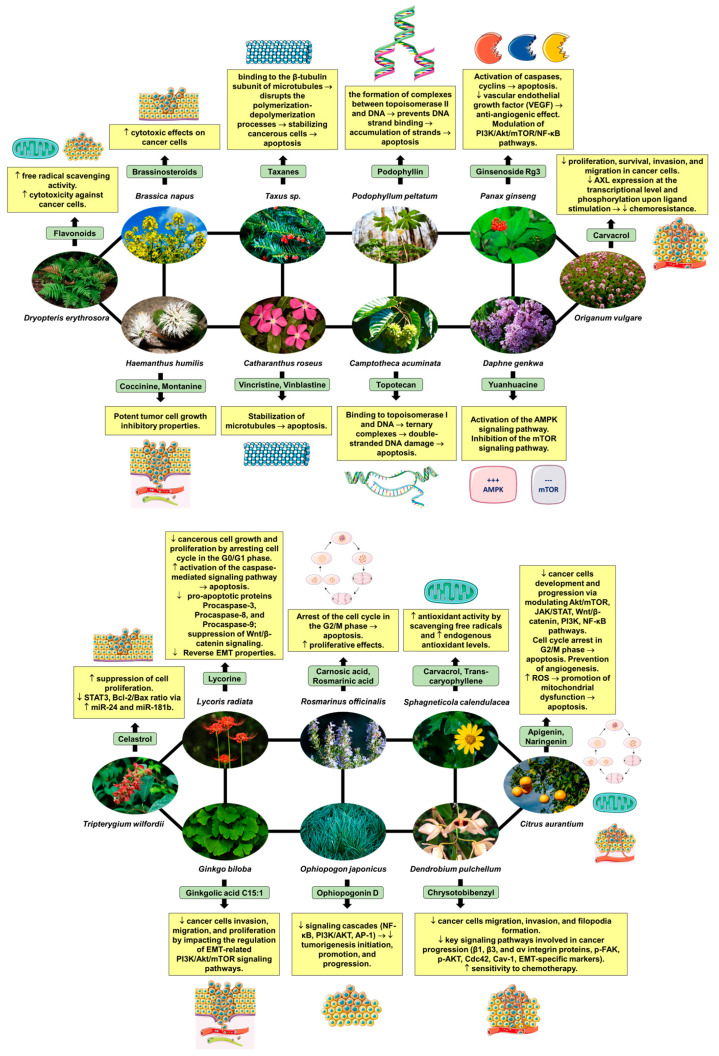
Mechanism of action of phytocompounds isolated from various medicinal plants with anticancer effects demonstrated in preclinical studies. Sources for the images and mechanisms of action of the plants depicted in Figure 1 will be detailed in the references section with respect to *Dryopteris erythrosora* (https://www.alamyimages.fr/photos-images/dryopteris-erythrosora.html?sortBy=relevant, accessed on 29 April 2024) [4], *Brassica napus* (https://www.alamyimages.fr/photos-images/brassica-napus.html?sortBy=relevant, accessed on 29 April 2024) [4,92], *Haemanthus humilis* (https://www.alamyimages.fr/photos-images/haemanthus-humilis.html?sortBy=relevant, accessed on 29 April 2024) [93], *Taxus* sp. (https://www.alamyimages.fr/photos-images/taxus-sp.html?sortBy=relevant, accessed on 29 April 2024) [94], *Catharanthus roseus* (https://www.alamyimages.fr/photos-images/catharanthus-roseus.html?sortBy=relevant, accessed on 29 April 2024) [95], *Podophyllum peltatum* (https://www.alamyimages.fr/photos-images/podophyllum-peltatum.html?sortBy=relevant, accessed on 29 April 2024) [96], *Camptoteca acuminata* (https://www.alamyimages.fr/photos-images/camptotheca-acuminata.html?sortBy=relevant, accessed on 29 April 2024) [97], *Panax ginseng* (https://www.alamyimages.fr/photos-images/panax-ginseng-nature.html?sortBy=relevant, accessed on 29 April 2024) [98,99,100,101], *Daphne genkwa* (https://www.alamyimages.fr/photos-images/daphne-genkwa-plant.html?sortBy=relevant, accessed on 29 April 2024) [102], *Origanum vulgare* (https://www.alamyimages.fr/photos-images/origanum-vulgare.html?sortBy=relevant, accessed on 29 April 2024) [103], *Trypterygium wilfordii* (https://www.alamyimages.fr/photos-images/Tripterygium-wilfordii.html?sortBy=relevant, accessed on 29 April 2024) [104], *Lycoris radiata* (https://www.alamyimages.fr/photos-images/lycoris-radiata.html?page=3&sortBy=relevant, accessed on 29 April 2024) [105], *Ginkgo biloba* (https://www.alamyimages.fr/photos-images/ginkgo-biloba.html?sortBy=relevant, accessed on 29 April 2024) [106], *Rosmarinus officinalis* (https://www.alamyimages.fr/photos-images/rosmarinus-officinalis.html?sortBy=relevant, accessed on 29 April 2024) [107], *Ophiopogon japonicus* (https://www.alamyimages.fr/photos-images/ophiopogon-japonicus.html?sortBy=relevant, accessed on 29 April 2024) [108], *Sphagneticola calendulacea* (https://www.alamyimages.fr/photos-images/Sphagneticola-calendulacea.html?sortBy=relevant, accessed on 29 April 2024) [109], *Dendrobium pulchellum* (https://www.alamyimages.fr/photos-images/dendrobium-pulchellum.html?sortBy=relevant, accessed on 29 April 2024) [110], and *Citrus aurantium* (https://www.alamyimages.fr/photos-images/citrus-aurantium.html?sortBy=relevant, accessed on 29 April 2024) [111]. The figures in the diagram were created using Servier Medical Art, licensed under Creative Commons Attribution 3.0 Unported License.

Figure 1 illustrates the intricate mechanism of action of phytocompounds found in medicinal plants that exhibit anticancer properties, particularly in the context of preclinical studies focusing on lung cancer. The diagram depicts various stages of the anticancer activity of these phytocompounds, starting from their interaction with specific molecular targets involved in cancer cell proliferation, survival, and metastasis. It also presents the main signaling pathways involved in tumor growth inhibition, apoptosis induction, and inhibition of angiogenesis in lung cancer cells. Overall, the diagram provides a visual representation of the multifaceted mechanisms through which phytocompounds from medicinal plants exert their anticancer effects in preclinical lung cancer models. The data presented in Figure 1 serve as a valuable resource for understanding the growing body of evidence supporting the use of medicinal plants and phytocompounds as potential adjuncts in the treatment of lung cancer, thereby highlighting their significance in the realm of oncological research.

## 5. Synergistic Effects between Natural Compounds and Chemotherapy in Lung Cancer

### 5.1. General Aspects of Synergism and Combination Therapy

Synergism, or pharmacological synergy, is the result of the interaction of combined components to produce new and different effects than individual components (this definition usually refers to the effects of whole plants and not just the active compounds). The synergy concept may overlap with the potentiation concept, which describes a synergic effect in which the combined effect of two drugs administered simultaneously is greater than the total effect of drugs administered individually [114,115,116].

The objective of synergism resides in the administration of associated drugs at diminished doses compared to a single administration of them in larger doses. Notwithstanding the dose reduction, the sought-after therapeutic effect should ideally approximate, at a minimum, that achieved with the drugs administered in isolation, all while mitigating the exacerbation of adverse reactions [115].

Combination therapy intricately intersects with the concept of synergy. Evolving combination therapy has demonstrated remarkable efficacy, particularly in addressing advanced pathologies such as cancer, hypertension, and asthma, thereby substantially enhancing the quality of life of afflicted patients [117]. In the context of cancer therapy, the association of two or more pharmacological agents with heightened efficacy, targeting either cancer cells or the implicated pathways, reinforces treatment protocols [118]. This integrated therapeutic approach exerts a salient positive influence on treatment outcomes, halting drug resistance by subjecting cancer cells to the concomitant and synergistic toxicities induced by the combined substances [15].

Over the last 20 years, the use of conventional medicines in combination with complementary and alternative medicine, including phytotherapy, gemmotherapy, pharmacognosy, ethnopharmacology, and homeopathy (mainly using herbal products), has become more common. In the case of natural compounds and conventional chemotherapeutic drugs, the goal is to improve the efficacy of the latter while reducing their toxicity [116].

Clinico-pharmacological studies have led to the conception that at least four mechanisms of synergistic effects may be involved: (a) synergistic multi-target effects (a compound, a mixture of compounds or a plant extract may act on different targets, not only as expected, i.e., on all cellular functional or structural constituents); (b) modulation of pharmacokinetic or physicochemical effects (change in the physicochemical properties, including solubility, of a compound or a mixture of compounds); (c) impairment of resistance mechanisms (presence of natural derivatives, which may antagonize the development of drug resistance); and (d) elimination or neutralization potential (a compound or plant extract may have the ability to eliminate or neutralize the toxic effect of a drug, thereby reducing or reversing its adverse effects) [116].

In the contemporary context, combination therapy has transitioned beyond its initial novelty. Yet, the true novelty lies in the revelation of novel additive interactions that confer supplementary advantages to modern medical interventions.

### 5.2. Synergistic Effects of Combination Therapy (Natural Compounds and Chemotherapy) in Lung Cancer

The combination of phytocompounds or plant extracts with complementary therapy is highly appreciated and used for the treatment of cancer because it offers a novel approach that can enhance the efficacy of treatment and reduce toxicity while also potentially overcoming drug resistance. By harnessing the unique properties of natural compounds and their ability to augment the effects of chemotherapeutic drugs, it could become possible to improve outcomes for lung cancer patients and pave the way for more effective and personalized treatment approaches in the future [4,119].

The combination of natural compounds with conventional chemotherapeutic drugs has shown promising results in the treatment of lung cancer. Preclinical studies have demonstrated that natural compounds can sensitize lung cancer cells to chemotherapeutic drugs, leading to enhanced anticancer activity [24,120,121,122]. Additionally, the translational implications of natural agents in cancer treatment have been highlighted, emphasizing their potential to overcome drug resistance when combined with conventional chemotherapy [123]. By further exploring and understanding the synergistic interactions between natural compounds and conventional chemotherapeutic drugs, it is possible to discover potential new treatment strategies for lung cancer [18].

Several studies have explored the synergistic interplay between plants and plant-derived compounds and chemotherapy agents. For example, the combination of the phytocompound emodin with the chemotherapies sorafenib, afatinib, cisplatin, paclitaxel, gemcitabine, and endoxifen showed synergistic effects with potentiated anticancer activities against lung adenocarcinoma and non-small cell lung cancer [4].

Table 3 provides a comprehensive overview of relevant preclinical studies that demonstrate the synergistic interaction between medicinal plants and phytocompounds in the treatment of lung cancer. The table presents a detailed summary of the various preclinical studies conducted in this area, highlighting the specific medicinal plants and phytocompounds that have shown potential synergistic effects in combating lung cancer. Each study is meticulously documented, including information on the experimental design, methodologies, and key findings. The table serves as a valuable resource for researchers and clinicians, offering a consolidated view of the preclinical evidence supporting the synergistic interaction between medicinal plants and phytocompounds in the context of lung cancer treatment. It provides a foundation for further investigation and potential translation into clinical applications, underscoring the significance of natural compounds in the development of novel therapeutic strategies for lung cancer.

#### 5.2.1. *Scutellaria baicalensis* and Cisplatin

The combination of the plant *Scutellaria baicalensis* (family Lamiaceae) and cisplatin, one of the first-line chemotherapeutics in the treatment of lung cancer, demonstrated synergistic anticancer effects and reduced cisplatin-induced adverse effects in the study by Huang et al. *Scutellaria baicalensis* has shown synergistic effects with cisplatin, inhibiting tumor growth both in vivo (C57BL/6J tumor-inoculated mouse model) and in vitro (Lewis lung carcinoma cells–LLC). The combination treatment of cisplatin and *Scutellaria baicalensis* significantly reduced tumor volume and weight, while also mitigating cisplatin-induced cachexia and renal tubular damage. The extract was able to inhibit cancer cell growth, induce mitochondrial damage, induce apoptosis, and arrest the cell cycle through various molecular pathways involving Bcl family genes, c-myc proto-oncogenes, CDK inhibitors, as well as ERK, mTOR, and NFκB pathways. These preclinical findings suggest a potential novel approach in the treatment of lung cancer through the synergistic interactions between natural compounds and conventional chemotherapeutic drugs [2].

#### 5.2.2. *Marsdenia tenacissima* and Gefitinib

The combination of the plant *Marsdenia tenacissima* (family Apocynaceae) and the chemotherapy gefitinib showed synergistic effects in the study by Han et al., inhibiting chemotherapy resistance by restoring sensitivity to gefitinib in resistant non-small cell lung cancer (NSCLC). In NSCLC patients treated with gefitinib, EGFR-activating mutations led to treatment resistance. The combination of *Marsdenia tenacissima* extract and gefitinib induced apoptosis in resistant cells through a synergistic effect using H460, H1975, and H292 cell lines. Flow cytometry results indicated that the combination treatment reduced EGFR downstream signaling pathways, including phosphorylation of PI3K/Akt/mTOR, c-Met, and ERK1/2, more effectively than each drug alone. This combination therapy offers a comprehensive approach to address resistance mechanisms and target key cancer progression signaling pathways in lung cancer [124].

#### 5.2.3. Zhen-Qi Sijunzi Decoction and Cisplatin

The combination of Zhen-Qi Sijunzi decoction (ZQ-SJZ) and cisplatin chemotherapy resulted in attenuation of cisplatin-induced toxicity and a prolonged survival rate in cachectic mice through the recovery of muscle atrophy, according to the study by Chen et al. The ZQ-SJZ decoction, consisting of *Panax ginseng*, *Atractylodes macrocephala*, *Poria cocos*, *Glycyrrhiza uralensis*, *Hedysarum polybotrys*, and *Ligustrum lucidum*, was administered to female C57BL/6 mice bearing Lewis lung carcinoma (LLC) cancer cells. Treatment with ZQ-SJZ alongside cisplatin resulted in the recovery of body weight, restoration of intestinal mucosal damage, and improved appetite in the mice. The combination therapy of ZQ-SJZ and cisplatin alleviated cisplatin-induced toxicity, prolonged the survival rate of cachectic mice, and reversed muscle atrophy by modulating mitochondrial metabolic function. In a C2C12 cell model, ZQ-SJZ inhibited cisplatin’s cytotoxic effect on myotubes, enhancing muscle strength and overall survival. This therapy offers a holistic approach to addressing cancer-induced cachexia by modulating myogenic protein levels, preventing muscle wasting, and improving mitochondrial function [126].

#### 5.2.4. *Panax ginseng* and Cisplatin

The combination of ginsenoside Rg3 (steroidal saponin) extracted from *Panax ginseng* (family Araliaceae) and the chemotherapeutic cisplatin can combat cancer cell chemoresistance in non-small cell lung cancer (NSCLC) by inhibiting programmed death ligand 1 (PD-L1), according to the study by Jiang et al. In the in vitro experimental phase, A549 and A549/DDP (cisplatin-resistant) human lung cancer cell lines were treated with varying concentrations of ginsenoside Rg3 (5–160 μg/mL). Treatment with 80 μg/mL and 160 μg/mL concentrations significantly reduced cell viability to below 50% in both cell lines, while lower concentrations (5–40 μg/mL) resulted in a less significant inhibition of cell viability, above 50%. Additionally, ginsenoside Rg3 (40 μg/mL) was found to enhance cisplatin cytotoxicity, decrease PD-L1 and Akt/NF-κB signaling pathways, and increase apoptosis in A549/DDP cells. It also potentiated the cytotoxicity effect of T-CD8+ cells by decreasing the PD-L1 expression [138]. Other studies have indicated its potential to improve chemotherapy efficacy and reverse tumor cell resistance in advanced NSCLC [127].

#### 5.2.5. *Vitis vinifera* and Paclitaxel

Resveratrol, a polyphenolic compound found in the fruits of the plant *Vitis vinifera* (family Vitaceae), has also been reported to enhance the efficacy of conventional chemotherapeutic drugs in lung cancer treatment. A study by Kong et al. demonstrated that resveratrol increased the sensitivity of lung cancer cells to paclitaxel, a commonly used chemotherapeutic drug, by promoting cell cycle arrest and apoptosis. The synergistic effect was attributed to the inhibition of the mTOR pathway and the downregulation of anti-apoptotic proteins, such as Bcl-2 and survivin [128].

#### 5.2.6. Apigenin and Cisplatin

Apigenin, a flavonoid abundantly found in citrus fruits, chamomile, and onions, exhibits properties that make it suitable for both preventing and treating cancer [86]. The combined effectiveness of apigenin with cisplatin in the context of lung cancer was evaluated through the MTT test. The inclusion of apigenin led to an increase in the apoptotic cell population. Furthermore, a reduction in cancer stem cells, which are associated with cisplatin resistance, was observed in a manner dependent on the expression of the tumor suppressor p53. The combination therapy was found to reduce the incidence of drug resistance and improve the overall survival rate of lung cancer patients [129].

#### 5.2.7. *Citrus aurantium* and Paclitaxel

The compound 5-demethylnobiletin, a member of the flavonoid class, is well-known for its antiproliferative properties, particularly in its ability to impede the proliferation of cancer cells. This bioactive compound is abundantly present in orange peels, making it a subject of interest in the field of cancer research [130]. Cytotoxicity evaluations were performed on lung cancer cells, where 5-demethylnobiletin was administered alongside the chemotherapeutic agent paclitaxel. The apoptosis test revealed an increase in cytotoxic effects, coupled with a simultaneous reduction in the adverse effects of paclitaxel. Based on their research findings, Tan et al. assert the presence of synergism and endorse the adoption of this combined regimen as a chemotherapeutic intervention [139]. Despite the recognized effectiveness of the compounds, their solubility presents a significant hurdle. Consequently, efforts were made to incorporate them into nanostructured lipid carriers functionalized with cetuximab. The combination index was computed to assess the synergy of the compounds, both in combination and individually, and the calculated index value for the combined groups was <1, signifying enhanced synergistic efficacy [131].

#### 5.2.8. Fisetin and Paclitaxel

An additional compound noted for its synergistic interaction with a chemotherapeutic agent is fisetin [132]. Fisetin, a naturally occurring flavonoid, is predominantly sourced from strawberries and it is also present in various fruits, vegetables, and nuts. It is widely acknowledged for its anti-inflammatory, antioxidant, and anti-angiogenic properties within the scientific domain [140]. The study by Klimaszewska-Wisniewska et al. aimed to assess the synergistic impact of co-administering fisetin with paclitaxel on lung cancer cells. The MTT assay was implemented at varying concentrations of both substances, revealing an inverse correlation between cell viability and fisetin dosage. Subsequent empirical validation post-experimentation demonstrated that the concurrent administration of these compounds induces cytotoxicity at lower paclitaxel doses when accompanied by fisetin. This observed phenomenon serves to alleviate the adverse reactions associated with the chemotherapy drug, resulting in a markedly superior effect on lung cancer cells compared to individual substance administration [132].

#### 5.2.9. *Hedera helix* and Cisplatin

Hederagenin, identified as a pentacyclic triterpene isolated from *Hedera helix* [91], has been substantiated to harbor therapeutic attributes akin to anti-inflammatory [141] and anticancer activities [142], characteristic of oleanane-type triterpenic acids [143]. The efficacy of hederagenin was evaluated in conjunction with cisplatin to ascertain its potential as a chemotherapy adjuvant. These assessments were grounded in its capacity to impede autophagy (a process pivotal to the disposal of damaged mitochondria) in cancer cells. After the administration of hederagenin alone and in combination with cisplatin on NCI-H1299 and NCI-H1975 cells, a significant improvement in the apoptosis of cancer cells was observed. The provided results indicate that hederagenin, in isolation, proficiently inhibits autophagy without significantly influencing cell proliferation. The noteworthy disparity emerged in the context of the hederagenin-cisplatin combination, leading to an early onset of apoptosis [133].

#### 5.2.10. *Brucea javanica* and Cisplatin

In a parallel line of inquiry, dehydrobruceine B, a quassinoid categorized as a triterpenic lactone, has been scrutinized for its inhibitory impact on cell proliferation in lung cancer. Originating from *Brucea javanica*, dehydrobruceine B has demonstrated a reduction in cellular viability, as evidenced by the MTT test on A549 and NCI-H292 lung cancer cells [134]. Ongoing research endeavors are directed toward refining this compound’s combinatory potential, with the overarching objective of determining its feasibility as an adjuvant in chemotherapy. Research findings have elucidated that dehydrobruceine B instigates a pronounced surge in the generation of reactive oxygen species (ROS) within cancer cells subjected to cisplatin treatment [135]. This intracellular escalation of ROS precipitates oxidative stress, constituting a pivotal molecular cue in the intricate process of apoptosis [144]. Moreover, it was revealed that dehydrobruceine B augments the perturbation of mitochondrial membrane potential. A decrease in MMP may also be linked to apoptosis [145]. Notably, when synergistically administered with cisplatin, dehydrobruceine B effectively instigates the depolarization of the mitochondrial membrane potential, further underscoring its intricate role in orchestrating cellular responses to apoptotic stimuli. Hence, investigations conducted by Shandong University have conclusively substantiated the synergistic efficacy of dehydrobruceine B in conjunction with cisplatin in the context of lung cancer [135].

#### 5.2.11. *Cucumis melo* and Cisplatin/Paclitaxel

In the context of further research on lung cancer, emphasis is placed on 2-deoxy-2-amine-cucurbitacin E (DACE), a derivative of cucurbitacin B [136]. Cucurbitacins belong to the class of tetracyclic triterpenoids found in the family Cucurbitaceae and are used in traditional medicine for their therapeutic properties, including antitumor effects [146]. In the study conducted by Marostica et al., the antiproliferative effects of DACE in combination with cisplatin and paclitaxel in lung cancer were assessed using the A459 cell line. The study exposed three cell lines to varying concentrations of DACE, cisplatin, and the DACE-cisplatin combination. The combination treatment showed the most significant reduction in cell proliferation compared to the individual administration of DACE and cisplatin. Similar results were observed when cisplatin was replaced with paclitaxel, with the combination of DACE and paclitaxel yielding the best outcomes. Although paclitaxel exhibited significant efficacy in inhibiting cell proliferation, it did not surpass the results obtained by combining both substances. These findings suggest that DACE may enhance the antitumor action of chemotherapeutic agents [136].

#### 5.2.12. *Curcuma longa* and Cisplatin

The synergistic effects of curcumin, a natural compound found in *Curcuma longa* (family Zingiberaceae), and cisplatin, a commonly used chemotherapeutic drug, in non-small cell lung cancer (NSCLC) were studied over the years. It was found that the combination of curcumin and cisplatin significantly enhanced the anti-tumor effects of cisplatin in NSCLC cells both in vitro and in vivo. The combination also reduced the toxicity of cisplatin on normal lung cells. Curcumin can sensitize NSCLC cells to the cytotoxic effects of cisplatin, making it a potential adjuvant therapy in the treatment of lung cancer [137,147].

**Figure 2:**
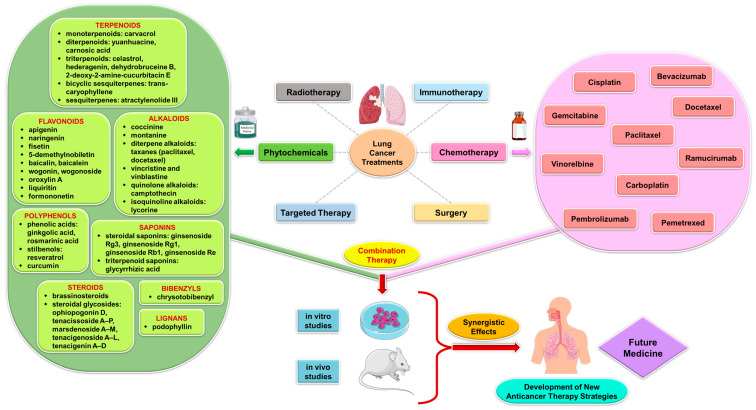
Synergistic interactions between phytocompounds from medicinal plants and conventional therapy in lung cancer treatment.

Figure 2 presents schematically the synergistic interactions between the primary phytocompound classes derived from medicinal plants and conventional therapy in the context of lung cancer treatment. This figure serves as a visual representation of the combined effects of phytochemicals and standard cancer treatments, showcasing how these compounds work together to enhance therapeutic outcomes in lung cancer patients. The phytocompound classes highlighted in the figure likely include alkaloids, flavonoids, terpenoids, and phenolic compounds, among others, which have been extensively studied for their anti-cancer properties [4]. By combining these natural compounds with traditional cancer therapies such as chemotherapy, radiation therapy, and targeted therapies, a synergistic effect can be achieved, leading to improved efficacy, reduced side effects, and potentially overcoming drug resistance mechanisms in lung cancer cells [52]. This integration of phytochemicals with conventional treatments represents a promising approach in the field of oncology, offering new avenues for enhancing the overall management of lung cancer.

## 6. Outline Perspectives

The synergistic interactions between natural compounds and conventional chemotherapeutic drugs in preclinical models of lung cancer present a promising avenue for further exploration in clinical settings. Future research should focus on translating these preclinical findings into clinical trials to evaluate the efficacy and safety of combination therapies. Additionally, the potential for personalized medicine approaches, where the selection of treatment is tailored to the genetic and molecular profile of individual patients, could be a significant perspective to consider. This could lead to more targeted and effective treatment strategies for lung cancer patients. Further investigation into the underlying mechanisms of the synergistic interactions identified in preclinical models is essential. Elucidating the molecular pathways and signaling cascades involved in the synergistic effects of natural compounds and chemotherapeutic drugs can provide valuable insights for the development of novel therapeutic targets. Moreover, the identification of biomarkers associated with treatment response and resistance to combination therapies could facilitate the development of predictive tools for patient stratification and monitoring of treatment outcomes.

Drug resistance and adverse side effects are major challenges in the treatment of lung cancer. The exploration of synergistic interactions between natural compounds and conventional chemotherapeutic drugs offers a potential strategy to overcome drug resistance and reduce the toxicity of standard chemotherapy regimens. Future perspectives should focus on investigating the ability of combination therapies to circumvent resistance mechanisms and minimize the adverse effects associated with traditional treatments. The integration of natural compounds with conventional chemotherapeutic drugs represents a bridge between conventional and complementary/alternative medicine approaches. This perspective opens up opportunities for interdisciplinary collaboration between researchers, clinicians, and practitioners of traditional medicine. Exploring the potential synergies between natural compounds and standard treatments aligns with the growing interest in integrative oncology and holistic patient care. As the field of combination therapies continues to evolve, it is crucial to address regulatory and ethical considerations associated with the use of natural compounds in conjunction with conventional chemotherapeutic drugs. Perspectives should encompass discussions on standardization, quality control, and safety assessments of natural compounds, as well as ethical considerations related to patient consent, information disclosure, and the integration of complementary approaches within the existing healthcare framework.

The perspectives outlined above provide a comprehensive outlook on the implications and future directions arising from the exploration of synergistic interactions between natural compounds and conventional chemotherapeutic drugs in preclinical models of lung cancer.

## 7. Conclusions

The study of synergistic interactions between natural compounds and conventional chemotherapeutic drugs has emerged as a novel approach to the treatment of lung cancer. The findings of numerous preclinical studies have demonstrated the potential of combined therapies to enhance the effectiveness of chemotherapy agents while reducing their toxic side effects. Natural compounds have shown promising results in sensitizing lung cancer cells to chemotherapy drugs by modulating various molecular pathways involved in tumor growth and progression. However, further research is needed to better understand the mechanisms behind these interactions and to determine the optimal combinations and dosages for clinical application. Nonetheless, the development of these synergistic approaches holds great promise for improving lung cancer treatment outcomes and highlights the importance of integrating traditional and alternative medicine in the fight against this deadly disease. Moreover, the development of standardized plant extracts and effective delivery systems will facilitate the translation of these findings into clinical applications. With the increasing prevalence of lung cancer worldwide and the limitations of current treatment options, the exploration of medicinal plants and phytocompounds as alternative therapeutics is crucial and holds immense potential for addressing the burden of this disease.

## Figures and Tables

**Table 1:** Overview of relevant preclinical studies evidencing the main phytocompounds used in lung cancer treatment.

Plant, Used Parts, Family	Extract Type	Phytocompounds	Study Type: In Vitro/In Vivo; Lung Cancer Cell Line	Dosage	Refs.
*Dryopteris erythrosora* (leaves and rhizomes), Dryopteridaceae	Ethanolic extract	Flavonoids	A549 lung cancer cell line (in vitro culture)	—	[4]
*Brassica napus* (pollen), Brassicaceae	—	Brassinosteroids (steroidal phytohormones)	A549 lung cancer cell line (in vitro culture)	—	[4,92]
*Haemanthus humilis* (bulbs), Amaryllidaceae	Aqueous acidic extracts	Coccinine and montanine (alkaloids)	A549 lung cancer cell line (in vitro culture)	1 to 50 μM	[93]
*Taxus* sp. (bark, leaves, seeds, roots), Taxaceae	—	Diterpene alkaloids, collectively called taxanes: paclitaxel, docetaxel, and cabazitaxel	—	—	[94]
*Catharanthus roseus* (herba–aerial parts), Apocynaceae	—	Vincristine and vinblastine (alkaloids)	—	—	[95]
*Podophyllum peltatum* (rhizome), Berberidaceae	—	Podophyllin (lignan)	—	—	[96]
*Camptotheca acuminata* (bark and stems), Nyssaceae	—	Topotecan (camptothecin derivative)	—	—	[97]
*Panax ginseng* (roots), Araliaceae	—	Ginsenoside Rg3 (steroidal saponin)	H1975 cell line (in vitro) and xenograft nude mouse model (in vivo)	—	[98,99,100,101]
*Daphne genkwa* (flower buds), Thymelaeaceae	Mixt extract (acetone:methanol:chloroform = 1:1:1)	Yuanhuacine (daphnane diterpenoid class)	human H1993 lung cancer cells (in vitro culture); H1993-implanted xenograft nude mouse model (in vivo)	0.5–1 mg/kg, once daily, for 21 days (in vivo)	[102]
*Aromatic plants* (volatile oil), mainly Lamiaceae	—	Carvacrol (monoterpenoid class)	human A549 and H460 lung cancer cells (in vitro culture)	—	[103]
*Tripterygium wilfordii* (roots), Celastraceae	—	Celastrol (pentacyclic quinine triterpenoid)	human A549 lung cancer cell line (in vitro culture)	3 and 4.5 μM	[104]
*Lycoris radiata* (bulbs), Amaryllidaceae	—	Lycorine (isoquinolinic compound)	human A549 and H460 lung cancer cell lines (in vitro culture); A549/Luc-implanted xenograft nude mouse model (in vivo)	10, 20 and 30 μM	[105]
*Ginkgo biloba* (leaves, pseudo-drupes, seeds), Ginkgoaceae	—	Ginkgolic acid C15:1 type (phenolic acids class)	human A549 and H1299 lung cancer cell lines (in vitro culture)	30 to 150 μM	[106]
*Rosmarinus officinalis* (leaves), Lamiaceae	Methanolic extract	Carnosic acid (phenolic diterpene) and rosmarinic acid (phenolic compound)	NCI-H82 human small cell lung carcinoma cell line (in vitro culture)	6.25 to 100 μg/mL	[107]
*Ophiopogon japonicus* (roots), Asparagaceae	—	Ophiopogonin D (steroidal glycoside)	H1299 and A549 lung cancer cell lines (in vitro culture)	1 to 10 μM	[108]
*Sphagneticola calendulacea* (leaves), Asteraceae	—	Carvacrol (monoterpenoidic compound) and trans-caryophyllene (bicyclic sesquiterpene)	C57BL/6 mouse animal model with B16F10 metastatic melanoma tumor xenograft (in vivo)	5, 10, 25, 50, and 100 μg/mL	[109]
*Dendrobium pulchellum* (stem), Orchidaceae	Ethanolic extract	Chrysotobibenzyl (bibenzylic compound)	lung cancer cell lines H460, H292, A549, and H23 (in vitro culture)	50 to 100 µM	[110]
Various plants, such as *Citrus aurantium* (Rutaceae) or *Matricaria recutita* (Asteraceae)	—	Apigenin and naringenin (flavonoids)	A549 and H1299 non-small cell lung cancer (NSCLC) cell lines (in vitro culture)	28.7 and 32.5 µM	[111]

**Table 2:** Chemical structures of key phytocompounds from medicinal plants with efficacy in preclinical lung cancer studies. The chemical structures of key phytocompounds were generated using KingDrawHD v1.4.5.-20230617 software.

Botanical Name of the Plant	Name of the Phytocompounds	Chemical Structures of the Phytocompounds
*Dryopteris erythrosora*	Flavonoids	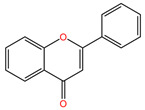
*Brassica napus*	Brassinosteroids	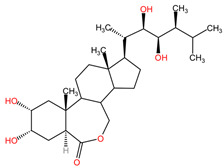
*Haemanthus humilis*	Coccinine	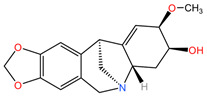
	Montanine	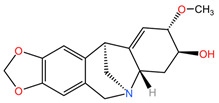
*Taxus* sp.	Paclitaxel	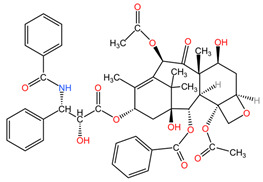
*Catharanthus roseus*	Vincristine	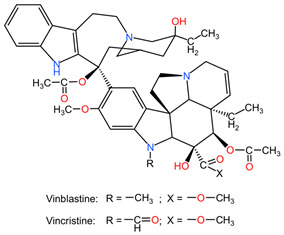
	Vinblastine	
*Podophyllum peltatum*	Podophyllin	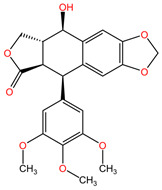
*Camptotheca acuminata*	Topotecan	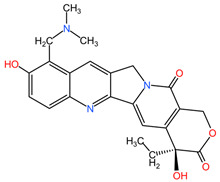
*Panax ginseng*	Ginsenoside Rg3	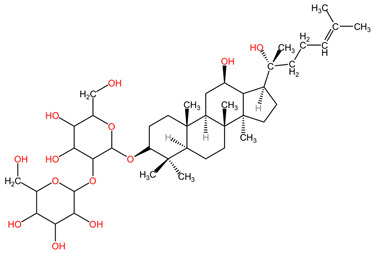
*Daphne genkwa*	Yuanhuacine	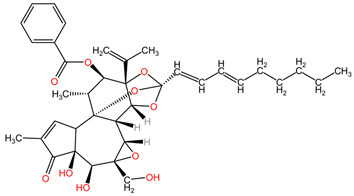
*Aromatic plants*	Carvacrol	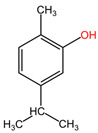
*Tripterygium wilfordii*	Celastrol	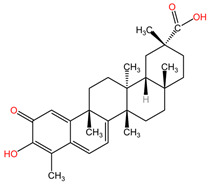
*Lycoris radiata*	Lycorine	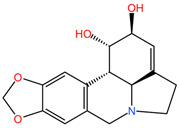
*Ginkgo biloba*	Ginkgolic acid C15:1 type	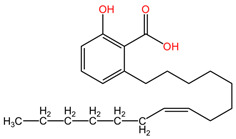
*Rosmarinus officinalis*	Carnosic acid	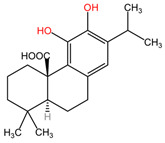
	Rosmarinic acid	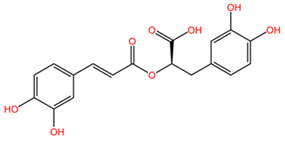
*Ophiopogon japonicus*	Ophiopogonin D	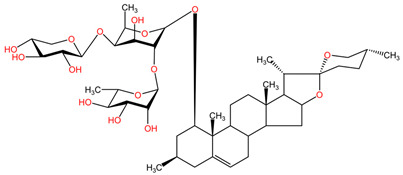
*Sphagneticola calendulacea*	Trans-caryophyllene	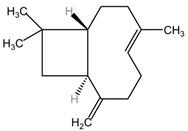
*Dendrobium pulchellum*	Chrysotobibenzyl	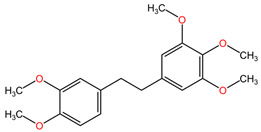
*Various plants, such as Citrus aurantium or Matricaria recutita*	Apigenin	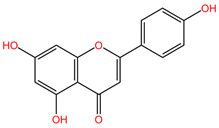
	Naringenin	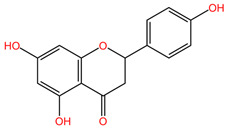

**Table 3:** Overview of relevant preclinical studies evidencing the synergistic interaction between medicinal plants and phytocompounds in lung cancer treatment.

Medicinal Plant, Family	Phytocompound(s)	Chemotherapeutic Drug	Experimental Model	Concentration	Effects	Refs.
*Scutellaria baicalensis* (Lamiaceae)	Baicalin (the major compound), baicalein, wogonin, wogonoside, oroxylin A	Cisplatin	C57BL/6J tumor-inoculated mouse model (in vivo) and Lewis lung carcinoma cells–LLC (in vitro)	*Scutellaria baicalensis* extract–orally, 300 mg/kg/day. Cisplatin–intraperitoneally, 3 mg/kg/day.	The plant extract effectively reduced cisplatin-induced adverse effects by inhibiting cancer cell growth, causing mitochondrial damage, apoptosis, and cell cycle arrest through various molecular pathways.	[2]
*Marsdenia tenacissima* (Apocynaceae)	C21 steroidal glycosides and their derivatives: tenacissoside A–P, marsdenoside A–M, tenacigenoside A–L, tenacigenin A–D	Gefitinib	human NSCLC cell lines: H460, H1975, and H292 (in vitro)	Gefitinib–1 µM. *Marsdenia tenacissima*–20–100 g crude drug (=20–100 mL).	Inhibition of chemotherapy resistance by restoring sensitivity to gefitinib in NSCLC. Reduced EGFR downstream signaling pathways, reducing phosphorylation of key signaling molecules involved in cancer cell survival and proliferation: PI3K/Akt/mTOR, c-Met, and ERK1/2.	[124,125]
Zhen-Qi Sijunzi decoction: *Panax ginseng*, *Atractylodes macrocephala*, *Poria cocos*, *Glycyrrhiza uralensis*, *Hedysarum polybotrys*, and *Ligustrum lucidum*	Glycyrrhizic acid (2.422 mg/g), Liquiritin (1.202 mg/g), Ginsenoside Rb1 (0.390 mg/g), Ginsenoside Rg1 (0.333 mg/g), Ginsenoside Re (0.251 mg/g), Salidroside (0.042 mg/g), Formononetin (0.015 mg/g), Atractylenolide III (0.002 mg/g).	Cisplatin	female C57BL/6 mouse model, injected intraperitoneally with Lewis lung carcinoma cancer cells (in vivo)	Cisplatin–5 mg/kg, intraperitoneal, 3 days/week. ZQ-SJZ–700 mg/kg, oral, 5 days/week.	Attenuation of cisplatin-induced toxicity and a prolonged survival rate in cachectic mice through the recovery of muscle atrophy. Combating muscle atrophy in lung cancer patients by modulating myogenic protein levels, preventing muscle wasting, and improving mitochondrial function.	[126]
*Panax ginseng* (Araliaceae)	Ginsenoside Rg3 (steroidal saponin)	Cisplatin	human lung cancer cell lines A549 and A549/DDP (cisplatin-resistant) (in vitro)	Ginsenoside Rg3–5, 10, 20, 40, 80, 160 μg/mL	Inhibition of cancer cell chemoresistance in NSCLC by blocking PD-L1 and Akt/NF-κB signaling pathways; potentiation of the cytotoxicity effect of T-CD8+ cells.	[127]
*Vitis vinifera* (Vitaceae)	Resveratrol (polyphenolic compound)	Paclitaxel	NSCLC cell line A549 (in vitro)	Resveratrol (5 µg/mL) + Paclitaxel (5 µg/mL); Resveratrol (5 µg/mL) + Paclitaxel (10 µg/mL).	Increased the sensitivity of lung cancer cells to paclitaxel treatment by inhibiting the mTOR pathway and the downregulation of anti-apoptotic proteins, such as Bcl-2 and survivin.	[128]
*Citrus sp*. (Rutaceae), *Matricaria recutita* (Asteraceae), *Allium cepa* (Amaryllidaceae)	Apigenin (flavonoid)	Cisplatin	NSCLC A549, H1299 cell lines (in vitro); a xenograft mouse model (in vivo)	Apigenin–25 µM; cisplatin–2.5 µM (in vitro). Apigenin–50 mg/kg; cisplatin–3 mg/kg (in vivo).	Apigenin improved the efficacy of cisplatin by boosting its ability to induce apoptosis in cancer cells while reducing cisplatin’s toxicity to normal cells. The combination therapy decreased the incidence of drug resistance and improved the overall survival rate of lung cancer patients.	[86,129]
*Citrus aurantium* (Rutaceae)	5-demethylnobiletin (flavonoid)	Paclitaxel	CL1–5 lung cancer cells (in vitro); nude mouse xenograft model (in vivo)	Paclitaxel–3.1, 6.3, 12.5, 25.0 nM + 5-DMN 10 μM; 5-DMN–1.56, 3.1, 6.3 12.5 μM + Paclitaxel 10 nM (in vitro). Paclitaxel–10 mg/kg; 5-DMN–3 mg/kg; 5- DMN + Paclitaxel–3 mg/kg + 10 mg/kg (in vivo).	The low concentrations of the combination treatment led to a reduction in cell viability and a concomitant increase in apoptosis by modulating the caspase pathway (caspase-3, caspase-8, and caspase-9 activities).	[130,131]
Various fruits, vegetables	Fisetin (flavonoid)	Paclitaxel	NSCLC A549 cell line (in vitro)	Fisetin–10, 20, 30, 40, 50 μM. Paclitaxel–0.1, 0.2, 0.3, 0.4, 0.5 μM.	The combination therapy upregulated the expression of proteins involved in apoptosis and autophagy, inducing mitotic catastrophe and autophagic cell death. It also increased the production of ROS and caused DNA damage, leading to cell death.	[132]
*Hedera helix* (Araliaceae)	Hederagenin (pentacyclic triterpene)	Cisplatin, Paclitaxel	NCI-H1299 and NCI-H1975 cell lines (in vitro)	Hederagenin–6 μM. Cisplatin, Paclitaxel– 2 μM.	Hederagenin enhances the anticancer properties of cisplatin and paclitaxel, leading to cell viability reduction and apoptosis in lung cancer cells. It suppresses autophagy and inhibits the mTOR signaling pathway, indicating its potential for cancer treatment.	[133]
*Brucea javanica* (Simaroubaceae)	Dehydrobruceine B (triterpenic lactone)	Cisplatin	A549 and NCI-H292 lung cancer cells (in vitro)	Dehydrobruceine B–1 μM. Cisplatin–9, 18 μM.	The combination therapy instigates the depolarization of the mitochondrial membrane potential, increasing the production of ROS and leading to oxidative stress and ultimately, cell death. Dehydrobruceine B can regulate the expression of proteins involved in the mitochondrial apoptotic pathway, leading to increased apoptosis in lung cancer cells treated with cisplatin.	[134,135]
Semisynthetic derivative of cucurbitacin B, isolated from *Cucumis melo* (Cucurbitaceae)	2-deoxy-2-amine-cucurbitacin E = DACE (tetracyclic triterpenoids)	Cisplatin, Paclitaxel	A459 lung cancer cell line (in vitro); xenograft female BALB/c nude mice (in vivo)	DACE 1 mg/kg + PTX 10 mg/kg	The combined treatment reduced tumor growth and proliferation, reducing residual viable tumor mass and inhibiting cell proliferation, making it less susceptible to drug resistance compared to individual treatments.	[136]
*Curcuma longa* (Zingiberaceae)	Curcumin (biphenolic compound)	Cisplatin	human lung cancer adenocarcinoma cells A549 and H2170 (in vitro)	41 μM curcumin + 30 μM cisplatin for A549 cells; 33 μM curcumin + 7 μM cisplatin for H2170 cells	The combination therapy significantly enhanced cisplatin’s anti-tumor effects, sensitizing NSCLC cells and reducing its toxicity on normal lung cells.	[137]

## Data Availability

The data are available at request from the corresponding author.
